# Inferior vena cava thrombosis—rope ladder sign

**DOI:** 10.1007/s10354-021-00886-y

**Published:** 2021-10-06

**Authors:** Sascha Meyer, Martin Poryo

**Affiliations:** 1 Department of Pediatrics and Neonatology, University Children’s Hospital of Saarland, 66421 Homburg, Germany; 2 Department of Pediatric Cardiology, University Children’s Hospital of Saarland, Homburg, Germany

**Keywords:** Congenital heart disease, Children, Rope ladder sign, Thrombosis, Univentricular heart/pathology

## Abstract

Congenital heart disease comprises one of the largest groups of congenital defects, affecting approximately 1% of births. Advances in pre- and postoperative critical care treatment as well as surgery and interventional procedures have improved survival rates, but treatment and long-term care of children with complex congenital heart disease remains challenging, and is associated with a number of complications.

Here, we report on a 17-month-old infant with congenital univentricular heart disease who devloped post-operatively inferior vena cava (IVC) thrombosis. IVC thrombosis was confirmed by a bedside contrast media study (X-ray) demonstrating collateral paravertebral circulation along the paravertebral sinuses bilaterally into the azygos and hemiazygos vein (“rope ladder sign“), with no contrast media detected in the IVC. The infant was subsequently started on aspirin and clopidogrel.

We report the case of a 17-month-old infant who was initially treated with Damus–Kaye–Stansel surgery and Blalock–Taussig shunt for hypoplastic left heart syndrome (stage I palliation). He was now admitted to our hospital for hemi-Fontan surgery (stage II palliation).

On postoperative ultrasonography, inferior vena cava (IVC) thrombosis was suspected. We subsequently performed a bedside contrast media study using the indwelling left femoral central catheter. Immediately after injection of contrast media, a conventional chest and abdominal X‑ray demonstrated drainage of the contrast agent along the paravertebral sinuses bilaterally into the azygos and hemiazygos vein (“rope ladder” appearance), with no contrast media detected in the IVC (Fig. 1). This finding confirmed thrombosis of the IVC. On conventional X‑ray, cardiac enlargement as well as metal clips after interventional closure of major aortic–pulmonary collateral arteries and a left-sided chest drainage following surgery were also noted (Fig. 1). The infant was subsequently started on aspirin and clopidogrel.

**Fig. 1:**
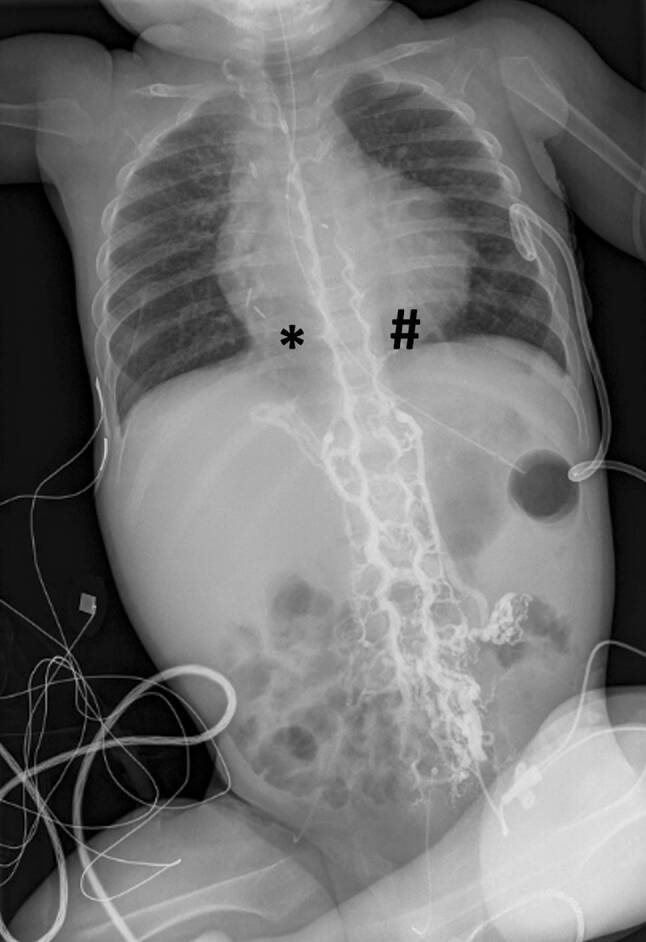
Conventional chest and abdominal X‑ray after contrast media injection demonstrating drainage of the contrast agent along the paravertebral sinuses bilaterally (“rope ladder” appearance) into the azygos (*asterisk*) and hemiazygos vein (*diamond*) with no contrast media in the inferior vena cava (IVC)

Congenital heart disease comprises one of the largest groups of congenital defects, affecting approximately 1% of births [1]. Advances in pre- and postoperative critical care treatment as well as surgery and interventional procedures have led to increased survival rates, but treatment and long-term care of children with complex congenital heart disease remains challenging [2].

Thrombosis is one of the most frequent complications affecting children with congenital heart disease, and it is associated with increased morbidity and mortality [3]. There is a multitude of both acquired and congenital risk factors for increased thrombotic risk, including complex pathologic anatomy, dilated atrium or ventricle, artificial valves, low flow phenomena (limited outflow/inflow), hypercoagulability, polycythemia, hyperviscosity, venous catheterization, the presence of an indwelling catheter, and surgery as well as infections. Monotherapy or combination therapy (anticoagulation with antiplatelets) and/or thrombolytic agents exist for different prophylactic or treatment indications in this high-risk cohort. Patients with single ventricle physiology—as our patient—are at a particular high risk for the development of thrombosis. Therefore, meticulous clinical monitoring and long-term medical prophylaxis is mandatory in these patients [1].

This case report demonstrates the feasibility of conventional X‑ray studies using contrast media in depicting collateral paravertebral circulation, thus, establishing a diagnosis of IVC thrombosis without resorting to more sophisticated and expensive imaging modalities.
